# Antibacterial and Biofilm Degradation Effects of Hungarian Honeys Linked With Botanical Origin, Antioxidant Capacity and Mineral Content

**DOI:** 10.3389/fnut.2022.953470

**Published:** 2022-07-13

**Authors:** Ágnes Farkas, Viktória Lilla Balázs, Tamás Kõszegi, Rita Csepregi, Erika Kerekes, Györgyi Horváth, Péter Szabó, Krisztián Gaál, Marianna Kocsis

**Affiliations:** ^1^Department of Pharmacognosy, Faculty of Pharmacy, University of Pécs, Pécs, Hungary; ^2^Department of Laboratory Medicine, Medical School, University of Pécs, Pécs, Hungary; ^3^János Szentágothai Research Center, University of Pécs, Pécs, Hungary; ^4^Department of Microbiology, Faculty of Science and Informatics, University of Szeged, Szeged, Hungary; ^5^Institute of Geography and Earth Sciences, Faculty of Sciences, University of Pécs, Pécs, Hungary; ^6^Research Institute for Viticulture and Enology, University of Pécs, Pécs, Hungary; ^7^Department of Plant Biology, Institute of Biology, University of Pécs, Pécs, Hungary

**Keywords:** honey, pollen spectrum, antioxidant capacity, mineral content, food-borne pathogens, antibacterial effects, biofilm degradation

## Abstract

The aim of the study was to assess the impact of four unifloral honeys on the food-borne pathogens *Pseudomonas aeruginosa* and *Staphylococcus aureus*, by analyzing the honeys’ antibacterial and biofilm degradation effects, as well as their antioxidant activity and element content. Linden and milkweed honeys represented light colored honeys, while goldenrod and chestnut honeys the darker ones. The botanical origin of the honeys and the relative frequency of their pollen types were established with melissopalynological analysis. The antioxidant capacities were calculated by two single electron transfer based methods (TRC – Total Reducing Capacity and TEAC – Trolox Equivalent Antioxidant Capacity) and a hydrogen atom transfer based assay (ORAC – Oxygen Radical Absorbance). The amount of four main macro- and two microelements was quantified. The antibacterial activity was determined by minimum inhibitory concentration (MIC) and membrane degradation assays. Furthermore, the biofilm degradation power of the samples was studied as well. The light colored linden honey with the lowest TRC and TEAC, but with the highest ORAC antioxidant activity and high element content showed the best antibacterial and biofilm degradation effects. Meanwhile, the dark colored chestnut honey with significantly higher single electron transfer based antioxidant capacities, with high element content, but lower ORAC showed significantly higher MIC and lower membrane degradation activity than linden honey. In case of biofilm degradation, both honey types gave similarly high inhibitory effect. Goldenrod honey was similarly effective regarding its MIC properties like chestnut honey, but had significantly lower antioxidant potential and ability to disrupt bacterial membranes and biofilms. Milkweed honey was the honey type with the lowest bioactivity and element content. The honeys, unequivocally characterized by their antioxidant characters and element content, displayed different antibacterial and biofilm degradation effects. In addition, some honey traits were found to be good predictors of the antimicrobial potential of honeys: ORAC assay showed correlation with the MIC values of both bacteria, and strict correlation was found between the mineral content and the antibiofilm activity of the studied honeys. Our studies indicate that unifloral honeys, such as linden and chestnut honeys, are plant-derived products with great potential as antimicrobial agents in food preservation, exhibiting remarkable antibacterial activity against food-borne pathogens.

## Introduction

Honey is an excellent source of bioactive constituents inherited mainly from the floral source. These plant secondary metabolites and minerals contribute to the biological properties, such as antioxidant activities and antibacterial effects of honey. Several studies confirmed that different honey types display significantly different biological activities (e.g., (1–3)). Thus, a key aspect before applying any kind of honey for health promoting and/or food preserving purposes, is the proper identification of the honey.

The tool for the accurate authentication of the botanical origin of honey samples is the time-consuming melissopalynological analysis. Identification of unifloral honeys is commonly based on establishing their characteristic pollen profile (1, 4), but physicochemical properties, color intensity, bioactive composition, etc., should also be taken into account (5–7). In case of unifloral honeys, there are specific organoleptic characters, based on which the honey can be identified with great certainty. However, in case of multifloral honeys, setting up the whole pollen profile would be advisable to establish their diverse botanical origin (8–10).

The color of honey is a good indicator of its polyphenolic and mineral content, which in turn determine the bioactivity of honey. Darker honeys tend to have enhanced properties (2, 11, 12), high macroelement content (13, 14) and the best biological activity (3). However, there are some exceptions, such as the light colored linden, sourwood and arbutus honeys with similarly high antioxidant activities like those of the dark honeys (1, 15). The antioxidant ability of plant-derived compounds is generally presented as their total antioxidant capacity (TAC) based on single electron transfer (SET) or hydrogen atom transfer (HAT) methods. The antioxidant activity and mineral content of several different honey types were summarized in the respective reviews of Martinello and Mutinelli (16) and Solayman et al. (14).

The health promoting properties of honey, allowing its topical use for the treatment of burns, wounds and skin disorders, as well as consuming honey as functional food, are largely based on its potent antibacterial and antibiofilm effects (17). The broad-spectrum antimicrobial activity of honey was presented in several studies. The components contributing to the antibacterial efficacy of honey include its sugar content, hydrogen-peroxide, polyphenol compounds and bee-defensin-1 (18). Polyphenols, with high levels in honey transmitted from the nectar, are representatives of the “non-peroxide antibacterial compounds,” which have been demonstrated to play a key role not only in the antimicrobial, but also in the antioxidant properties of honey (11, 18, 19). The antioxidant and antibacterial activities of several honey types were compared in reports from different countries, among others Brazil (10, 20), Chile (21), Australia (6) and Algeria (3). Studies on honey extracts of the Indonesian *Apis cerana* bee (22), or the Bornean stingless bee (23) related the antioxidant and antibacterial activities of these honeys to their polyphenolic content. Tsavea et al. (24) investigated the antibacterial activity and quality parameters of Greek pine honeydew honeys, suggesting multiple mechanisms of antibacterial activity.

The antioxidant and antimicrobial activity of honeys can be exploited in functional foods and can have a potential in food preservation. Honey was found to be effective on food-borne pathogens, such as *Staphylococcus aureus* and *Pseudomonas aeruginosa* (25–27). *S. aureus* is a gram-positive bacterium, which is one of the most frequently occurring pathogenic bacteria that can be found in the natural environment, which can cause infections and various inflammations. Moreover, it can pollute food and agricultural products like wheat, corn, and rye as well, which results in food poisoning (28). The heat-stable enterotoxins can cause foodborne illnesses (29). Foods that are not cooked after handling, such as sliced meats, puddings, pastries, and sandwiches, are especially risky if contaminated with *S. aureus* (30). *P. aeruginosa* is a gram-negative ubiquitous opportunistic bacterium. Those who have weaker immune system are more prone to get infected by this bacterium, which often stands in the background of the hospital-acquired acute and systemic infections (31). In addition, *P. aeruginosa* can cause contamination of meat and milk products (32). Many food-borne pathogens, including *S. aureus* and *P. aeruginosa* have the ability to create biofilms. The biofilm formation of these bacteria can provide protection against physical eradication, chemical elimination and disinfectants as well, which ability can cause food-borne diseases and spoilage (33).

To assess the impact of commonly available Hungarian honeys on food-borne pathogens, four unifloral honeys were selected, based on our previous results (1, 9). Selected honey types included the light colored linden, milkweed, the medium dark goldenrod and the dark chestnut honeys, whose SET based antioxidant capacity was positively correlated with their color. Furthermore, ORAC activity and the level of specific macro- and/or microelements were found to be reliable markers of the above honey types, in addition to their pollen spectrum. The purpose of our study was (1) to establish the effect of these selected honey types on the food-borne pathogens *P. aeruginosa* and *S. aureus*, (2) to reveal some of the underlying factors that may contribute to their antimicrobial effects, and (3) to improve the understanding of the nutritional values of these honeys. To the best of our knowledge, such a complex analysis of the antioxidant activity, mineral content and antibacterial properties of these Hungarian honeys has not been published yet.

## Materials and Methods

### Samples

The honey samples were purchased from three local apiaries in Hungary, in the year 2020; milkweed honeys originated from the Southern Great Plain area, while linden, goldenrod and chestnut honeys were harvested in the Southwest Transdanubium area. They were stored at room temperature (20–21°C) in the dark for a maximum of three weeks. For each honey type (Table 1), measurements were carried out on 3 parallel samples; altogether, 12 honey samples were analyzed.

**TABLE 1:** Sensory characteristics and color of analyzed honey samples.

Nr.	Honey Type, Plant Name	Sensory Characteristics (Odor and Consistency)	ABS_450–720_ (mAU)
1	Linden, *Tilia* spp.	Light amber, strong odor, fine granulated, semisolid	275 ± 5
2	Milkweed, *Asclepias syriaca*	Light yellowish amber, intense flower-like odor, liquid, viscous	308 ± 3
3	Goldenrod, *Solidago gigantea*	Amber, moderately intense odor, semisolid, fine granulated	563 ± 3
4	Chestnut, *Castanea sativa*	Amber with reddish tone, strong odor, liquid, viscous	764 ± 2

*Each code number in the first column represents three biological replicates (n = 3) of honey samples.*

### Melissopalynological Analysis

The botanical origin of honey samples was confirmed with microscopic pollen analysis, following the modified method of (34). Ten gram of thoroughly stirred honey was mixed with 20 mL distilled water, vortexing the mixture with Combi-spin FVL-2400N (Biocenter Ltd., Szeged, Hungary). Samples were centrifuged (3,000 rpm, 10 min) with a Neofuge 15R centrifuge (Lab-Ex Ltd., Budapest, Hungary). After decanting the supernatant, 10 mL distilled water was added to the sediment, and this mixture was centrifuged again (3,000 rpm, 5 min). To the sediment 0.25 mL distilled water was added and vortexed. From this pollen suspension 20 μL was pipetted on microscope slides previously placed on a heating plate (OTS 40, Tiba Ltd., Gyõr, Hungary) set to 40°C. After mounting pollen preparations in Kaiser’s glycerol jelly with fuchsine (Merck Life Science Ltd., Budapest, Hungary), pollen grains were studied with a Nikon Eclipse E200 light microscope equipped with a Michrome 20MP CMOS digital camera (Auro-Science Consulting Ltd., Budapest, Hungary). Microphotos were captured using Capture 1.2 at 400 × magnification. Counting at least 500 pollen grains from each honey sample, the botanical source was identified at plant species, genus or family level. The relative frequency of pollen types was calculated as the percentage of the total number of pollen grains.

### Determination of Color Intensity (ABS_450_)

Color intensity was determined following the protocol of (35). Honey solutions (50% w/v) were prepared with 45–50°C water, sonicated for 5 min, then filtered (0.45 μm pore size, Agilent Technologies, Milan, Italy). Absorbance was measured at 450 and 720 nm with a Shimadzu UV-1800 spectrophotometer (Shimadzu Schweiz GmbH, Reinach, Switzerland). Color intensity was calculated as the difference between absorbance at 450 and 720 nm, and results were expressed as milli-absorbance unit (mAU).

### Determination of Total Reducing Capacity

Total reducing capacity was measured using the Folin–Ciocalteau method as reported by Singleton et al. (36) with minor modifications. Honey samples (0.1 g) were dissolved in 1 mL distilled water. To 0.5 mL of this solution 100 μL of 10% Folin–Ciocalteau reagent was added, followed by 300 μL distilled water and 400 μL 6% Na_2_CO_3_ solution. Following a 20-min incubation period, the absorbance was determined at 760 nm. The results were expressed as mg of gallic acid per kg of honey (mg GAE kg^−1^). Gallic acid solutions in the range of 50 to 200 μg mL^−1^ were used as standard to establish the calibration curve. All chemicals were obtained from Merck Life Science Ltd., Budapest, Hungary.

### Determination of Trolox Equivalent Antioxidant Capacity

The trolox equivalent antioxidant capacity (TEAC) assay, which is based on the generation of ABTS radical cation (ABTS^•^+), was performed according to the method of Re et al. (37) and Stratil et al. (38), with slight modifications. ABTS^•^+ was produced by mixing ABTS stock solution (7 mM of ABTS dissolved in distilled water) with 2.45 mM K_2_S_2_O_8_ (final concentration) and diluted with PBS (pH 7.4) until the absorbance was 0.70 ± 0.005 at 734 nm. Then 20 μL aliquots of varying concentrations of the 50% aqueous honey extracts were allowed to react with 80 μL of ABTS^•^+ (7 mM). Following 20 min incubation in the dark, at room temperature, absorbance was measured at 734 nm by the Perkin Elmer EnSpire Multimode plate reader. Trolox was used as standard. All measurements were carried out in five independent experiments with three technical replicates.

### Oxygen Radical Absorbance Capacity

The oxygen radical absorbance capacity ORAC assay was conducted following the procedure described by (39, 40). In brief, a fluorescein working solution (400 nmol L^−1^, Merck Life Science Ltd., Budapest, Hungary) and the 2, 20 – azobis (2-amidinopropane) dihydrochloride (AAPH) oxidant (400 mmol L^−1^, Merck Life Science Ltd., Budapest, Hungary) dissolved in 75 mmol L^−1^ potassium phosphate buffer (mixture of KH_2_PO_4_ and K_2_HPO_4_, Reanal Labor, Budapest, Hungary) at pH 7.5 were prepared freshly before the measurements. Trolox standards were prepared in potassium phosphate buffer (0–160 μmol L^−1^). Into each well of optical plates (Perkin Elmer), 25 μL of the blank/standard/sample and 150 μL of fluorescein solution was pipetted and the mixture was incubated for 30 min in the dark at 37°C. Next, 25 μL AAPH solution/well was injected by the automated injector of a Biotek Synergy HT plate reader (BioTek Instruments, Winooski, VT, United States) previously warmed up to 37°C. The fluorescence intensities were monitored for 80 min (490/520 nm wavelengths) at 2 min intervals. The area under each curve (AUC) was obtained using the software of the reader providing the total sum of the individual digital data of the corresponding fluorescence signals. The antioxidant capacity values were expressed as μmol Trolox equivalent (TE) g^−1^ honey.

### Inductively Coupled Plasma Atomic Emission Spectrometry

Inductively coupled plasma atomic emission spectrometry measurements of eight elements were performed using an ICPE-9000 instrument (Shimadzu, Kyoto, Japan). The operating parameters were as follows: radio frequency power, 1.20 kW; plasma gas, 10.0 L min^−1^; auxiliary gas, 0.60 L min^−1^; carrier gas, 0.70 L min^−1^; and view direction, axial. Preceding the elemental analysis, honey samples were pretreated using a Multiwave 3,000 (Anton Paar GmbH, Graz, Austria) microwave system, in which 1 g of each honey sample was treated in three steps: 300 W for 5 min, 1,000 W for 5 min and 1,400 W for 20 min. The instrument was calibrated using inorganic reference standards for a single element (BDH Prolabo Chemicals, VWR International Kft., Debrecen, Hungary). Quality control was assured using a high-purity multielement standard solution containing 25 elements (HPS, RK Tech Kft., Budapest, Hungary). A recovery test was undertaken by spiking rape honey with 20 ppm of the ICP multielement standard mixture. Recoveries for the eight elements ranged from 93.8 to 111.5%. All analyses were carried out in triplicate. Detection limits (LOD) were as follows: 15.0 mg kg^−1^ for K, 10.0 mg kg^−1^ for Ca, 2.0 mg kg^−1^ for Mg, 1.5 mg kg^−1^ for P, 1.0 mg kg^−1^ for B, and 0.1 mg kg^−1^ for Cu, Mn, and Zn.

### Cultivation of Test Bacteria

The antibacterial effect of honey samples was determined on *Pseudomonas aeruginosa* ATCC 27853 and *Staphylococcus aureus* ATCC 25923. Test bacteria were grown in 100 mL BHI (Brain Heart Infusion, Sigma Aldrich Ltd., Hungary). Each bacteria were incubated in a shaker incubator (C25 Incubator Shaker, New Brunswick Scientific, Edison, New Jersey, United States) at 37°C and at a speed of 60 rpm for 12 h (41). The bacterial suspensions were diluted with clear BHI to the appropriate concentrations for each assay.

### Broth Microdilution Test

The minimum inhibitory concentrations (MIC) were determined with broth microdilution method. Microtiter plates (96-wells) were used to perform this assay. From each bacterium solution (10^5^ cfu/mL) 100 μL was measured to the wells. From honey samples 20, 30, 40, 50, 60, and 70% (w/v) stock solutions were prepared. It was taken into consideration that during the assay the concentrations of honey solutions were halved. To prepare the solutions with concentrations 20-50%, the honey samples were diluted in BHI, with the procedure described above. In case of 60 and 70% solutions, the bacterial suspension was used to dilute the honey samples. From these solutions 100 μL was added to the treated wells. After incubation (24 h, 37°C) absorbance was measured at 600 nm with a microtiter plate reader (BMG Labtech, SPECTROstar Nano, Budapest, Hungary). The negative control was the clear BHI, the positive control was the untreated bacterial suspension. The average of the six replicates was calculated and then the mean of the negative control was subtracted from the value obtained. Absorbance lower than 10% of the positive control samples, i.e., growth inhibition of 90% or more, was considered as the MIC value (42).

### Biofilm Degradation Activity

In order to prove the biofilm degradation capacity of each honey sample, crystal violet (CV) assay was used (43). The bacterial biofilms were prepared in 96-well microtiter plate. 200 μL of bacterial culture (10^8^ cfu/mL) was added into each well; then, the microtiter plate was incubated at 37°C for 4 h. After the incubation time, the non-adherent cells were washed with physiological saline solution. The honey samples were used in MIC/2 concentrations for the treatments. After the treatments, the microtiter plates were incubated again at 37°C for 24 h. Then the adherent cells were fixed with methanol (15 min). The biofilms were dyed with 0.1% crystal violet solution for 20 min. The redundant dye was removed. 33 w/w% of acetic acid was added to each well. The absorbance was measured (595 nm) with a microtiter plate reader (BMG Labtech SPECTROstar Nano, Budapest, Hungary). The biofilm degradation activity of the honey samples was calculated and demonstrated in terms of inhibitory rate according to the equation: Inhibitory rate = (1 *-* S/C) × 100% (C and S were defined as the average absorbance of control and sample groups, respectively).

### Membrane Degradation Study

The release of cellular material was examined in *P. aeruginosa* and *S. aureus*. The absorbance of 1 mL bacterial suspension containing 10^8^ cfu/mL in PBS (phosphate buffer saline) was measured at 260 nm. The bacterial cells treated with honey were suspended for 1 h in PBS containing 20, 40, and 60% (w/v) concentrations of honey samples. Control cells were suspended in PBS without honey treatment. As positive control 90% solution of honey samples was used. In order to study the kinetics of membrane degradation, the bacterial cells suspended in PBS containing 60% honey were treated for different periods of time: 0, 10, 20, 40, 60, and 90 min. After each treatment, cells were centrifuged (Neofuge 15R, Lab-Ex Ltd., Budapest, Hungary) at 11,107 rpm for 2 min, and the absorbance of the supernatant was determined at 260 nm with Metertech SP-8001 (Abl&e-Jasco Ltd., Budapest, Hungary) spectrophotometer. The results were expressed in percentage values, which were compared to the untreated cells (44).

### Scanning Electron Microscopy

Scanning electron microscopy was used to investigate the structural modifications of biofilms and the degradation of bacterial membrane after treatment with honey samples. For biofilm formation, 5 ml of *P. aeruginosa* and *S. aureus* culture (10^8^ cfu/mL) was added into sterilized bottles. Sterile coverslips were placed in the bottle and served as the attaching surface for the cells. The plates were incubated for 4 h at 37°C, then the planktonic cells and BHI were removed, and plates were rinsed with physiological saline. For treatment of biofilms, 5 mL honey samples (MIC/2 concentration) were added. The untreated coverslips were used as control. After incubation (24 h, 37°C), the supernatant was removed, and the bottles were washed with physiological saline. The preparation of the samples for electron microscopy was performed with 2.5% glutaraldehyde for 2 h at room temperature (23°C) to fix the biofilm formation. After dehydration of biofilms, different ethanol concentrations (50, 70, 80, 90, 95, and 98%) were used at room temperature for 2 × 15 min. Finally, t-butyl-alcohol: absolute ethanol solutions in 1:2, 1:1, and 2:1 ratios were added to the samples (each for 1 h, room temperature). After that the samples were dehydrated with absolute t-butyl alcohol for 2 h at room temperature. The samples were stored at 4°C for 1 h and freeze-dried overnight. Samples were coated with a gold membrane and observed with JEOL JSM IT500-HR scanning electron microscope (Jeol Ltd., Tokio, Japan) (42).

### Statistical Analysis

All measurements were completed on three biological replicates of four honey types. Statistical analyses were carried out using Excel^®^ (Microsoft Corp., Redmond, WA, United States) and the PAST software package version 3.11 (45) at a 5 or 1% significance level (*p* < 0.05, *p* < 0.01), after normality checking with the Shapiro–Wilk test. For the correlation matrix, moreover, the 0.1% (*p* < 0.001) significance level was used to indicate the greater significance of the differences. Data were expressed as means ± standard deviations (SD). Pairwise comparisons were performed with Student’s *t*-tests. Interactions between the measured parameters were investigated with Pearson’s rank correlation using PAST.

## Results

### Sensory Characteristics, Color and Pollen Analysis of Honey Samples

Evaluation of sensory characteristics (color, odor and consistency), absorbance determination and detailed melissopalynological analysis were carried out to identify the honey samples and to confirm their floral origin (Tables 1, 2). The unifloral origin of our linden honey sample was supported by the sensory characteristics and the high relative frequency (70%) of *Tilia* pollen. Milkweed honey was evaluated as unifloral honey, supported by its sensory characteristics and color intensity. Since honeybees are not able to harvest the large-size pollinia of milkweed, species specific pollen grains are not present in the honey. Our milkweed honey sample contained *Brassica* pollen as its dominant (>70%) pollen type, however, the color and consistency did not correspond to the sensory characteristics of rape honey. Color and consistency of goldenrod honey, and *Solidago* pollen as the dominant pollen type of this honey, confirmed its unifloral origin. The dark amber color and liquid consistency of the chestnut honey sample supported its unifloral origin, together with more than 80% *Castanea* pollen.

**TABLE 2:** Pollen spectrum of the studied honeys.

Honey type	Pollen type – Relative frequency (%)								
	*Tilia*	*Solidago*	*Castanea*	*Brassica*	*Robinia*	Asteraceae	Apiaceae	Lamiaceae	Other
Linden	69.7	26.1	–	3.2	–	1	–	–	–
Milkweed	–	–	–	71.0	1.4	0.7	0.7	0.4	25.8
Goldenrod	2.9	78.4	-	–	–	15.7	–	–	3
Chestnut	5.1	–	83.2	8.9	–	–	–	–	2.8

*Dominant pollen >45%, secondary pollen 16–45%, important minor pollen 3–15%, minor pollen <3% of the pollen grains counted.*

### Total Antioxidant Capacities of Honeys

Combination of two SET based – TRC, TEAC – and a HAT based – ORAC – methods were used to determine the bioactivity of honeys studied (Table 3). TRC distinguished the light colored honeys from the goldenrod and chestnut honeys, and also the latter two from each other. The distinctive power of TEAC was more limited than that of the TRC. The IC_50_ values of the TEAC analysis were the highest for linden honey (130.34 ± 12.86 IC_50_ mg mL^−1^), which means that its antioxidant power was significantly lower than that of the other honeys, supported also by its TRC value. Similarly to linden honey, the Folin-reactivity of milkweed honey was low, but its TEAC value (78.53 ± 5.84 IC_50_ mg mL^−1^) indicated significantly higher antioxidant capacity. In case of chestnut honey (533.76 ± 20.30 mg GAE kg^−1^), its average TRC was about twice as high as that of the goldenrod honey (255.63 ± 23.22 mg GAE kg^−1^), but their TEAC values did not differ significantly from each other. ORAC values differentiated all four honey samples, with surprisingly high value for the light colored linden honey (44.33 ± 5.38 μmol TE g^−1^), and with the lowest one for the other light colored honey, the milkweed honey (12.62 ± 1.14 μmol TE g^−1^).

**TABLE 3:** Total antioxidant capacities of the honey samples.

Nr.	Honey types	TRC (mg GAE kg^−1^)	TEAC (IC_50_ mg mL^−1^)	ORAC (μ mol TE g^−1^)
1	Linden	115.67 ± 13.74 ^a^	130.34 ± 12.86 ^a^	44.33 ± 5.38 ^a^
2	Milkweed	149.86 ± 30.84 ^a^	78.53 ± 5.84 ^b^	12.62 ± 1.14 ^b^
3	Goldenrod	255.63 ± 23.22 ^b^	64.29 ± 8.62 ^b^	15.37 ± 1.42 ^c^
4	Chestnut	533.76 ± 20.30 ^c^	71.52 ± 7.20 ^b^	20.18 ± 0.97 ^d^

*TRC—Total Reducing Capacity; TEAC—Trolox Equivalent Antioxidant Capacity; ORAC—Oxygen Radical Absorbance Capacity. Data are means ± standard deviations of three independent determinations (n = 3). Data in the same column with different superscripted letters mean significant differences among various honeys according to Student’s t-test (p < 0.01).*

### Multielement Analysis of Honeys

The main four macro- and two microelement contents of the honey samples were summarized in Table 4. The K content of linden and chestnut honeys was 4-5-times higher than that of milkweed and goldenrod honeys. The Ca content was relatively high in linden honey and low in milkweed honey. High P and Mg content characterized the chestnut honey, while milkweed honey had the lowest Mg content. Regarding the total element content of the honeys, linden (1,089.89 mg kg^−1^) and chestnut (1,134.88 mg kg^−1^) honeys contained significantly higher quantity of mineral elements than milkweed (247.67 mg kg^−1^) and goldenrod (464.76 mg kg^−1^) honeys. In case of the two selected microelements, extremely high Mn content characterized the chestnut honey, and high B content the goldenrod honey.

**TABLE 4:** Element content of the studied honey samples.

Nr.	Honey types	K (mg kg^−1^)	Ca (mg kg^−1^)	P (mg kg^−1^)	Mg (mg kg^−1^)	B (mg kg^−1^)	Mn (mg kg^−1^)
1.	Linden	861.34 ± 48.57^a^	161.06 ± 22.31^a^	39.62 ± 3.07^a^	23.28 ± 1.8^a^	2.63 ± 0.09^a^	1.95 ± 0.40^a^
2.	Milkweed	157.84 ± 11.42 *^b^*	39.31 ± 14.53^b^	35.59 ± 7.51^a^	11.00 ± 3.07^b^	3.82 ± 0.33^b^	0.10 ± 0.01^b^
3.	Goldenrod	280.34 ± 62.55^b^	105.90 ± 24.46^c^	43.99 ± 1.19^a^	28.12 ± 4.42^ac^	6.18 ± 0.64^c^	0.24 ± 0.11^b^
4.	Chestnut	906.49 ± 6.17^a^	120.32 ± 1.25^c^	60.46 ± 2.37^b^	32.57 ± 0.50^c^	4.05 ± 0.07^b^	10.99 ± 0.04^c^

*Data are means ± standard deviations of three independent measurements (n = 3). Data in the same column with different superscripted letters mean significant differences among various honeys according to Student’s t-test (p < 0.01).*

### Broth Microdilution Assay and Biofilm Degradation Study

**FIGURE 1:**
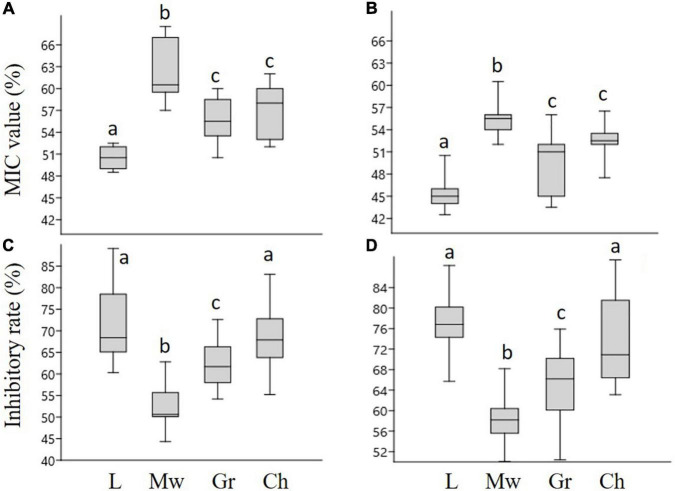
The minimum inhibitory concentration (MIC) values and inhibitory rates of honey samples, against *P. aeruginosa* **(A,C)**, and *S. aureus* **(B,D)**, respectively. L-linden honey, Mw-milkweed honey, Gr-goldenrod honey, Ch-chestnut honey. Different lower case letters above the boxes indicate significant differences among various honeys according to Student’s *t-*test (*p* < 0.01).

The MIC values of honey samples were determined by microdilution assay (Figures 1A,B). Linden honey showed the highest antibacterial activity with MIC values of 50.5 and 45.5% for *P. aeruginosa* and *S. aureus*, respectively. The highest MIC values, meaning the lowest activity, were measured in case of milkweed honey against both *P. aeruginosa* (62%) and *S. aureus* (55.5%). There was no statistical difference between the MIC values of goldenrod and chestnut honey against either bacterium. Biofilm degradation assay (Figures 1C,D) revealed high inhibitory rates in case of linden and chestnut honeys associated with the median inhibitory rates ranging between 68.5% (chestnut honey – *P. aeruginosa*) and 76.9% (linden honey – *S. aureus*). The milkweed and goldenrod honey samples produced significantly lower antibacterial activity; the lowest values belonged to the milkweed honey. The results clearly showed that each honey sample was effective against both bacteria, furthermore *S. aureus* was more sensitive than *P. aeruginosa*. Regarding the honey types, linden honey proved to be the most active sample, while milkweed honey was the least active one.

**FIGURE 2:**
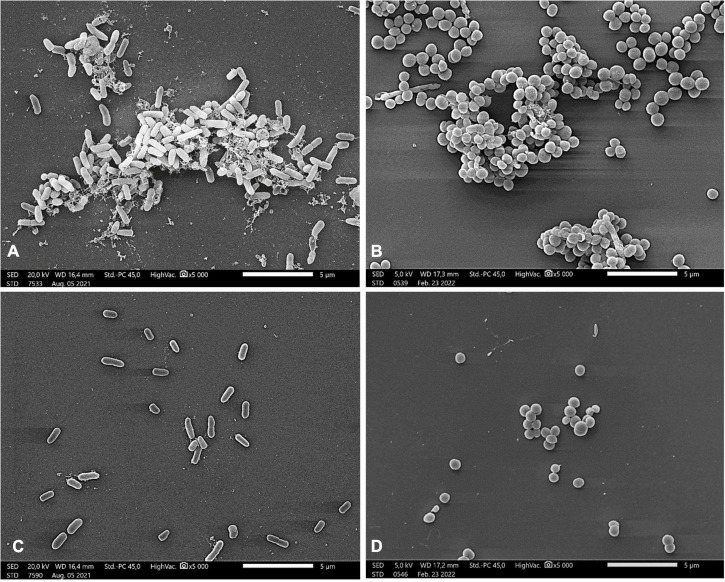
Scanning electron microscopic images of *P. aeruginosa* **(A,C)** and *S. aureus* **(B,D)** Control samples of bacterial strains **(A,B)**; treatment with 25.25% (w/v) and 22.75% (w/v) linden honey in case of *P. aeruginosa* and *S. aureus*, respectively **(C,D)**. Scale bar = 5 μm.

The high inhibitory effect of linden honey on biofilm formation of bacterial strains was illustrated in Figure 2. In case of both bacteria, the images of the control samples captured the characteristic morphological elements of a mature, three-dimensional biofilm (Figures 2A,B). The treatment with MIC/2 value of linden honey resulted that the cells attached to the surface, but they did not form biofilm-specific structures (Figures 2C,D).

### Membrane Degradation Study

In order to explore the mode of action of honey samples, the kinetics of bacterial membrane degradation was studied by measuring the degree of bacteriolysis of *P. aeruginosa* and *S. aureus* cells (Table 5). Different concentrations (0-90%) of honey samples were used. The 20% solutions did not cause any lysis in bacterial cells, while the 40 and 60% solutions were active. *S. aureus* responded more sensitively to the treatment than *P. aeruginosa*. In accordance with the results of biofilm degradation assay, the linden honey samples showed the best activity against both bacteria.

**TABLE 5:** The effect of honey solutions at different concentrations on the release of cellular material from *P. aeruginosa* and *S. aureus.*

Concentration (%)	Lysis of *P. aeruginosa* cells (%)				Lysis of *S. aureus* cells (%)			
	Linden	Milkweed	Goldenrod	Chestnut	Linden	Milkweed	Goldenrod	Chestnut
40	31.8 ± 3.0^a^	14.6 ± 2.9^b^	20.3 ± 1.6^c^	26.0 ± 3.1^d^	47.8 ± 2.6^a^	25.2 ± 3.5^b^	33.8 ± 1.5^c^	40.5 ± 2.0^d^
60	41.7 ± 1.5^a^	22.9 ± 1.3^b^	26.0 ± 1.7^c^	37.9 ± 1.4^d^	66.4 ± 3.8^a^	29.5 ± 6.3^b^	38.9 ± 2.2^c^	58.9 ± 4.3^d^
90	100	100	100	100	100	100	100	100

*Data are the release values presented in percentage vs. total ± SD (n = 6). Data in the same row with different superscripted letters indicate significant differences among various honeys according to Student’s t-test (p < 0.5).*

A time course lysis with 60% (*w*/*v*) honey solutions was also performed to examine the kinetics of bacterial membrane degradation (Table 6). The released cellular material was measured from 10 min to 90 min. The results showed that bacterial cell lysis was induced after 40 min incubation. Milkweed honey had the lowest activity compared to the other honey samples. Linden and chestnut honey samples were the most effective in case of both bacteria. The highest membrane degradation was detected at 90 min, in case of linden and chestnut honey against *S. aureus*.

**TABLE 6:** Kinetics of 260-nm absorbing material release from *P. aeruginosa* and *S. aureus* treated with 60% (*w/v*) honey solutions.

Time (min)	Lysis of *P. aeruginosa* cells (%)				Lysis of *S. aureus* cells (%)			
	Linden	Milkweed	Goldenrod	Chestnut	Linden	Milkweed	Goldenrod	Chestnut
40	28.9 ± 2.5^a^	10.0 ± 2.1^b^	16.7 ± 1.8^c^	26.7 ± 2.7^a^	45.6 ± 2.9^a^	17.6 ± 3.7^b^	21.1 ± 1.0^c^	43.4 ± 4.7^a^
60	41.7 ± 1.5^a^	22.9 ± 1.3^b^	26.1 ± 1.7^c^	37.9 ± 1.4^d^	66.4 ± 3.8^a^	29.5 ± 6.3^b^	38.9 ± 2.2^c^	58.9 ± 4.3^d^
90	72.1 ± 6.5^a^	44.3 ± 2.7^b^	53.1 ± 3.1^c^	65.7 ± 2.2^a^	86.4 ± 3.7^a^	53.3 ± 3.5^b^	60.2 ± 3.8^c^	73.0 ± 4.8^d^

*Data are the release values presented in percentage vs. total ± SD (n = 6). Data in the same row with different superscripted letters indicate significant differences among various honeys according to Student’s t-test (p < 0.5).*

**FIGURE 3:**
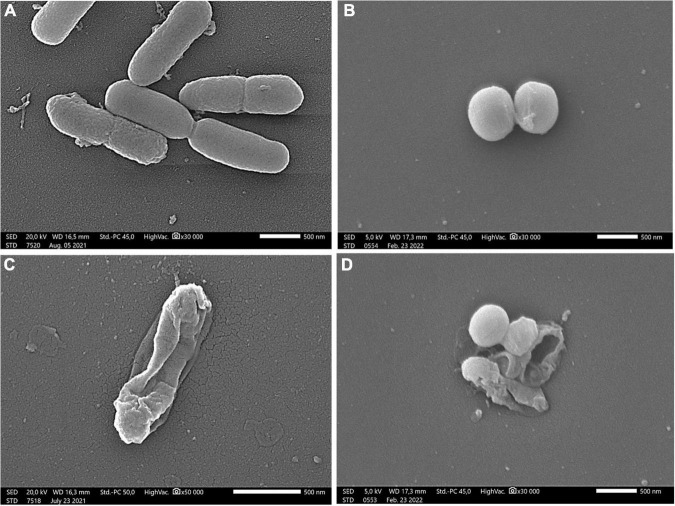
Scanning electron microscopic images of *P. aeruginosa* **(A,C)** and *S. aureus* **(B,D)** Control samples of bacterial strains **(A,B)**; treatment with 60% (w/v) linden honey **(C,D)**. Scale bar = 500 nm.

The membrane degradation effect of linden honey is clearly visible in Figure 3. The honey treatment caused morphological damage in the bacterial cells, disrupting the cell membrane, which in turn resulted in release of the cellular material.

### Correlation Analysis

The data matrix of color (absorbance), antioxidant, antibacterial, antibiofilm values and mineral contents was analyzed by Pearson’s correlation (Table 7). The following significant relationships were found. Correlation was obtained between color and the SET based methods (TRC and TEAC), between TEAC and ORAC. As expected, color and TRC did not show correlation with ORAC. Color and TRC assay were the strongest predicting factors regarding P, Mg and Mn content of the honey samples, while TEAC and ORAC were those of Ca and B content. Furthermore, the correlation between ORAC and K was also established. Regarding the antibacterial effect, ORAC showed correlation with the MIC values of both bacteria, while TEAC only with those of *P. aeruginosa*. In addition, all of the minerals, except B, showed strict positive correlation with the biofilm degradation values of both bacteria.

**TABLE 7:** Correlation matrix (Pearson’s correlation coefficients) of color, antioxidant, broth microdilution, biofilm degradation and macroelement parameters in Hungarian honeys.

Variable	Color	TRC	TEAC	ORAC	MIC_*Pa*	MIC_*Sa*	Bf_*Pa*	Bf_*Sa*
TRC	0.955***							
TEAC	−0.590*	−0.492						
ORAC	−0.412	−0.338	0.939***					
MIC_*Pa*	0.255	0.333	−0.634*	−0.711**				
MIC_*Sa*	0.248	0.372	−0.526	−0.603*	0.672*			
Bf_*Pa*	0.503	0.509	0.248	0.466	−0.486	−0.461		
Bf_*Sa*	0.483	0.579*	0.326	0.537	−0.337	−0.189	0.885***	
K	0.306	0.436	0.527	0.665*	−0.364	−0.243	0.800**	0.946***
Ca	0.113	0.123	0.659*	0.808**	−0.753**	−0.500	0.709**	0.763**
P	0.872***	0.906***	−0.299	−0.105	0.099	0.237	0.677**	0.695**
Mg	0.771***	0.717***	−0.065	0.126	−0.375	−0.135	0.760***	0.735***
B	0.485	0.258	−0.725**	−0.661*	0.102	0.108	−0.115	−0.322
Mn	0.769**	0.897***	−0.137	0.015	0.252	0.253	0.625*	0.779**

TRC—Total Reducing Capacity; TEAC—Trolox Equivalent Antioxidant Capacity; ORAC—Oxygen Radical Absorbance Capacity; MIC_Pa/Sa – MIC of P. aeruginosa/S. aureus; Bf_Pa/Sa – Biofilm inhibitory effect of P. aeruginosa/S. aureus; Significant at * p < 0.05, ** p < 0.01, *** p < 0.001.

## Discussion

Honey quality control is a major issue preceding its application for medical purposes or as functional food. Recently, (17) review drew attention to honey quality assurance, particularly in the case of medical-grade honeys, urging to revise the current qualitative tools within the European Union. A major strength of our study is the extensive assessment of selected European honey types, focusing not only on identification traits (color, sensory characteristics, pollen spectrum) and quality parameters (antioxidant capacity, mineral content), but also establishing a connection with their antibacterial and antibiofilm effects. Our current study confirmed the reliable quality parameters of linden, milkweed, goldenrod and chestnut honeys, supporting our previous observations (1, 9). As a starting point we selected the current honey samples based on the general observation that the color, antioxidant capacity and mineral content of honey were in positive correlation (1, 9, 46–48). In our case, the light colored linden honey was an exception with low TRC and TEAC, but high ORAC and mineral content. The remarkably high percentage of *Tilia* pollen in linden honey – which is typically under-represented in European honeys, with a mean relative frequency of 23% (49) – supported its unifloral origin. The similarly light colored milkweed honey had relatively low antioxidant parameters and mineral content, furthermore this honey type can be identified only by its typical sensory characters due to lack of *Asclepias* pollen in the honey. The dominance of rape pollen in milkweed honey did not influence its typical characters. In addition, our previous study revealed that in the Central European region true unifloral rape honeys often contain even higher percentage, more than 80% *Brassica* pollen (50). The color of the goldenrod honey sample corresponded to the previously described amber color of honey originating from *Solidago gigantea* (9, 51, 52). The high percentage of *Solidago* pollen in this sample supported its unifloral origin, completed with the measured antioxidant and mineral parameters, which were a bit higher than those of the milkweed honey. In the chestnut honey sample, the relative frequency of *Castanea* pollen remained a bit below the 90% required in many European laboratories in the case of this strongly over-represented pollen type (49), but its quality parameters and the extremely high Mn content unequivocally identified this honey. Furthermore, establishing statistically sound correlations among the antioxidant capacity and mineral content of honeys, supported our previous findings: strict positive correlation was found among color, TRC and P, Mg; between ORAC and K; no correlation between ORAC and TRC (9). However, the correlation between ORAC and other antioxidant methods may depend on the honey types studied. Bridi et al. (53) demonstrated that the ORAC-pyrogallol red index of quillai (*Quillaja saponaria*) honey was not correlated to the total polyphenolic content (TRC in our study), but highly correlated to the flavonoid content. In contrast, (10) revealed significant correlation between ORAC and total polyphenolic content.

When establishing the antimicrobial potential of honey, evaluation of its effect on biofilm-formation or on mature biofilms is an important aspect, because bacterial cells in biofilms are more resistant compared to their planktonic form. Several studies demonstrated the strong biofilm inhibitory potential of honey (17), which depends, however, on the honey type. Our previous study (54), conducted with respiratory tract bacteria, revealed differences in the antibacterial and antibiofilm activity of black locust, linden, and sunflower honeys, the highest activity shown by linden honey. Our current study also supported the antibacterial power of all honeys studied, with linden honey highlighted.

One of the most investigated honeys with high quality parameters, the manuka honey, exerted high antibiofilm activity against both food-borne bacteria involved in our study (27). In addition, honeys from other botanical sources showed similar or even higher activity, such as Polish multifloral and buckwheat honeys (25), or Slovakian blossom and honeydew honeys (17). Eucalyptus honey inhibited biofilm formation of *P. aeruginosa* and *S. aureus* by 40 and 60%, respectively (26). In our study, the inhibition rate of linden and chestnut honeys were even higher than 70%. The major antibacterial compound in many honeys is H_2_O_2_, but different bee or plant-derived factors seem to contribute to the antibiofilm effect of honey, among others defensine-1, osmolarity and protein fractions (26, 55). According to Brudzynski (56), dark colored honeys often produce higher amounts of H_2_O_2_ than light-colored ones. However, Farkasovska et al. (57) found weak or no correlation between the H_2_O_2_ content of linden honey and its antibacterial activity. In accordance with this observation, in our study the light-colored linden honey had similar antibiofilm activity as the dark-colored chestnut honey, furthermore both honeys contained remarkable amounts of minerals. The correlation analysis revealed a significant relation between the inhibitory effect and several minerals of the honeys.

The antibacterial effectiveness of honeys depends on the complex interaction of both participants, on the honey type and the bacterial species as well. The antibacterial and antibiofilm activity of different honey types against different bacteria are well documented in the literature (17, 18), including some studies on significant food-borne pathogens. The antibacterial potential of the dark colored buckwheat honey (58) and Greek honeys from Mount Olympus against *P. aeruginosa* and *S. aureus* was investigated and compared with that of manuka honey (59). The Chilean ulmo (*Eucryphia cordifolia*) honey showed greater antibacterial activity against methicillin-resistant *S. aureus* isolates than manuka honey, and similar effect against *P. aeruginosa* and *Escherichia coli* using agar diffusion method (60). Bucekova et al. (61) observed that the antibacterial activity of Slovakian commercial honeys against *S. aureus* is not uniform. Similarly, Hunter et al. (6) established differences in the antibacterial activity of different Australian honeys, depending on the bacterial species. All samples were effective as an inhibitory agent against *S. aureus*, MIC_50_ concentrations ranging from 0.078 to 5.82% (w/v), whereas the lowest concentration to inhibit the growth of *P. aeruginosa* was 25% (w/v). Lower sensitivity of *P. aeruginosa* to honey treatments was observed in other studies, as well. This bacterium was shown to exhibit antibiotic resistance to honey at lower concentrations (62), the reason for which might be its Gram-negative structure (63).

Differences in the sensitivity of Gram-positive bacteria vs. Gram-negative ones were observed in several studies. Gram-positive bacteria were more sensitive to Hungarian, Algerian and Greek honeys (3, 24, 54), compared to Gram-negative bacteria. However, in other studies conducted with Greek blossom honeys and Brazilian honey samples, Gram-positive bacteria were more resistant than the Gram-negative ones (20, 64). Furthermore, Liu et al. (19) found that the honey from *Bidens pilosa* had greater antibacterial activities against Gram-positive bacteria, including *Staphylococcus intermedius* and *Streptococcus alactolyticus*, and Gram-negative bacteria including *Citrobacter koseri* and hemolytic *E. coli*, than did the other honeys. In our study, all of the Hungarian honey types were more effective against the Gram + *S. aureus*, than the Gram- *P. aeruginosa*, in accordance with buckwheat and manuka honeys (58).

Focusing on the potent antibacterial role of the components hiding in this natural product, several researchers have concluded that the major antibacterial factors are hydrogen peroxide, catalase, and glucose oxidase, ahead of the non-peroxide compounds. Supporting this assumption, the amber dark colored honey sample among several other Brazilian honey samples, with the highest SET based antioxidant activity and high phenol and phenolic acid content did not show the best antibacterial activity, demonstrating that other compounds (for example hydrogen peroxide) may be more related to the inhibition of bacteria (20). In contrast, the honey samples from the semiarid Brazilian region, with the highest phenolic contents presented the lowest MIC values against the tested bacterial strains, which supported the main role of the non-peroxide compounds (polyphenolics and flavonoids) on the antibacterial scene (10). According to Alygizou et al. (65) observation, dark honeys are known to have higher antibacterial potential, which is partly connected to the H_2_O_2_ value. However, Tsavea et al. (24) did not observe correlation between hydrogen-peroxide concentration and the antibacterial activity of Greek pine honey samples. In our case, the high antibacterial and antibiofilm activities should be connected with high ORAC value. We did not find correlation between ORAC and TRC, maybe because this HAT based antioxidant method measures a bit different antioxidant group compared with the SET based ones, which generally correlate with total phenolic content. It was supported also by Bridi et al. (53), furthermore they have found connection between ORAC and flavonoid content of the honeys studied. The presence of higher amounts of flavonoids in Brazilian honeys, or in stingless bee honeys from Borneo could also contribute to their stronger antimicrobial activity (10, 23). In our study we hypothesized that the high antibacterial and antibiofilm activity of linden and chestnut honeys may be due at least partially to the higher level of their non-peroxide compounds, mainly flavonoids. Our preliminary measurements indicated significant differences between various honey types in the concentration of some phenolic compounds, e.g., chlorogenic acid, gentisic acid, hesperetin, kaempferol, p-syringaldehyde, pinobanksin, quercetin, quercitrin and taxifolin (data not shown). However, we could not establish a direct relationship between the concentration of these compounds and the honey’s biological activity.

Other studies, comparing quality, antioxidant and antibacterial properties of honeys, revealed further connections. Antibacterial activity of the Australian honey samples was associated with the antioxidant characteristic measured by FRAP (Ferric Reducing Antioxidant Power) method, and their phenolic content contributed to both antioxidant and antibacterial effects (6). Deng et al. (58) compared the biochemical properties, antibacterial and cellular antioxidant activities of the dark colored buckwheat and manuka honeys. Both honey types contained abundant minerals, buckwheat honey having even higher contents of Fe, Mn and Zn. The antibacterial activity of buckwheat honey against *P. aeruginosa* and *S. aureus* was comparable with that of manuka honey. Chilean honey samples could be characterized based on their highly variable antioxidant and antibacterial activity (21). The study of Algerian honeys revealed highly significant correlations between the inhibition zone diameters of *Salmonella typhi* and *Staphylococcus aureus* and antioxidant activity, as well as phenolic and flavonoid content (3). In addition, they concluded that the level of bioactive compounds, as well as the antioxidant and antibacterial activities of honeys depended on their botanical and geographical origin rather than their monofloral or polyfloral nature.

## Conclusion

The honeys, unequivocally characterized by their antioxidant characters and element content, displayed different levels of antibacterial and biofilm degradation activities. As a novel finding, promising antibiofilm activity was observed in case of our linden and chestnut honeys. Some honey traits were found to be good predictors of the antimicrobial potential of honeys: ORAC assay showed correlation with the MIC values of both bacteria, and strict correlation was found between the mineral content and the antibiofilm activity of the studied honeys. Our studies indicate that certain types of unifloral honeys, such as linden and chestnut honeys, are plant-derived products with great potential as antimicrobial agents in food preservation, exhibiting remarkable antibacterial activity against food-borne pathogens.

## Data Availability Statement

The original contributions presented in the study are included in the article/supplementary material, further inquiries can be directed to the corresponding author/s.

## Author Contributions

MK, ÁF, and VB contributed to the conception and design of the study. VB, RC, and EK performed the laboratory investigations. PS and VB collaborated in SEM studies. KG organized the database. MK performed the statistical analysis. MK and VB wrote the first draft of the manuscript. GH, TK, and ÁF wrote the sections of the manuscript. All authors contributed to manuscript revision, read, and approved the submitted version.

## Conflict of Interest

The authors declare that the research was conducted in the absence of any commercial or financial relationships that could be construed as a potential conflict of interest.

## Publisher’s Note

All claims expressed in this article are solely those of the authors and do not necessarily represent those of their affiliated organizations, or those of the publisher, the editors and the reviewers. Any product that may be evaluated in this article, or claim that may be made by its manufacturer, is not guaranteed or endorsed by the publisher.
